# Modeling of the thermal properties of SARS-CoV-2 S-protein

**DOI:** 10.3389/fmolb.2022.953064

**Published:** 2022-09-27

**Authors:** Ziyuan Niu, Karin Hasegawa, Yuefan Deng, Ziji Zhang, Miriam Rafailovich, Marcia Simon, Peng Zhang

**Affiliations:** ^1^ Department of Applied Mathematics and Statistics, Stony Brook University, Stony Brook, NY, United States; ^2^ Mathematics, Division of Science, New York University, Abu Dhabi, United Arab Emirates; ^3^ Department of Materials Science and Chemical Engineering, Stony Brook University, Stony Brook, NY, United States; ^4^ Department of Oral Biology and Pathology, Stony Brook University, Stony Brook, NY, United States

**Keywords:** SARS-CoV-2, S-protein, molecular dynamics, thermodynamics, conformational state

## Abstract

We calculate the thermal and conformational states of the spike glycoprotein (S-protein) of SARS-CoV-2 at seven temperatures ranging from 3°C to 95°C by all-atom molecular dynamics (MD) µs-scale simulations with the objectives to understand the structural variations on the temperatures and to determine the potential phase transition while trying to correlate such findings of the S-protein with the observed properties of the SARS-CoV2. Our simulations revealed the following thermal properties of the S-protein: 1) It is structurally stable at 3°C, agreeing with observations that the virus stays active for more than two weeks in the cold supply chain; 2) Its structure varies more significantly at temperature values of 60°C–80°C; 3) The sharpest structural variations occur near 60°C, signaling a plausible critical temperature nearby; 4) The maximum deviation of the receptor-binding domain at 37°C, corroborating the anecdotal observations that the virus is most infective at 37°C; 5) The in silico data agree with reported experiments of the SARS-CoV-2 survival times from weeks to seconds by our clustering approach analysis. Our MD simulations at µs scales demonstrated the S-protein’s thermodynamics of the critical states at around 60°C, and the stable and denatured states for temperatures below and above this value, respectively.

## 1 Introduction

The SARS-CoV-2 engulfs the world, even in tropical countries (Sarkar et al., 2020; Tosepu et al., 2020), and it remains infectious for weeks at 3°C–4°C in the cold supply chain (Dai et al., 2020; Rizou et al., 2020). Recent studies confirmed the negative correlation between the spread of the COVID-19 and climate (Sobral et al., 2020; Tosepu et al., 2020); moreover, the instantaneous reproduction number of SARS-CoV-2 is closely related to the temperature (Rubin et al., 2020).

The biological experiments with more laboratory details verified that coronaviruses are thermolabile. For example, SARS-CoV is inactivated after 75°C heat for 15 min, and MERS becomes inactivated after 65°C heat for 1 min (Darnell et al., 2004; Leclercq et al., 2014). These impacts are also apparent in the recent laboratory studies about SARS-CoV-2. It remains infectious in aerosols at 21°C–23°C for 3–24 h (Van Doremalen et al., 2020). The SARS-CoV-2 remains active at 37°C for at least 24 h but inactive after 60°C heat for 15 min (Wang et al., 2020). When the incubation temperature reaches 70°C, the virus is inactive within 5 min (Chin et al., 2020). These confirm that SARS-CoV-2 gradually loses viability with increasing temperature. The data available for understanding is still sparse, and handling these clinical specimens poses a biosafety risk to laboratory professionals and workers (Wang et al., 2020). It is necessary to introduce more computational simulations.

Understanding the biochemical and thermolabile properties of SARS-CoV-2 requires a comprehensive understanding of the structure at the atomic level. The S-protein is one of the coronavirus’s initial and largest structural proteins (Li, 2016). The outer membrane S-protein, similar with the genome to SARS-CoV’s S-protein (Ahmed et al., 2020), is the primary host interaction protein with host cell targets such as ACE2 and is critical for cell adherence and pathogenicity (Pöhlmann et al., 2012; Heise et al., 2018). The temperature dependence of the SARS-CoV-2 characteristics, notably for S-protein, is interesting in medical prediction because ambient temperatures first alter the structure of the virus protein membrane. The S-protein has two domains: the S1 domain is on top and comprises the portion that interacts directly with host cell receptors; the S2 domain, in contrast to the S1 subunit, forms the stalk of the S-protein (de Groot et al., 1987), facilitating virion fusion with cellular membranes (Walls et al., 2020). The receptor binding domain (RBD) is responsible for binding to ACE2, which is the initial step for entry into target cells (Lan et al., 2020). *In vitro* binding studies confirmed that the RBD on SARS-CoV-2 binds to ACE2 with a low nano band affinity, indicating a crucial functional component in the S1 domain that induces the binding to ACE2 (Tian et al., 2020; Walls et al., 2020). The RBD of the S1 domain undergoes hinge-like conformational changes, transitioning between the closed and open state (Heise et al., 2018). The SARS-CoV-2 may withstand a higher temperature than SARS-CoV, and RBD-ACE2 binding for SARS-CoV-2 is more temperature-sensitive than SARS-CoV (He et al., 2020). MD simulations also demonstrate a temperature-dependent binding affinity of SARS-CoV-2 to the ACE2 (Zhou et al., 2021) also provided the preliminary structural states of S-protein by 200 ns at 10°C–50°C (Rath and Kumar, 2020). At the outermost of the protein, N-terminal domain (NTD) is increasingly exposed. The NTD and RBD sections are more versatile, allowing for structural adaptation to host receptors (Verkhivker, 2020). The structure and binding sites of such S-proteins are well established, while other characteristics, especially the stability under external factors, such as temperature, remain elusive. Moreover, due to the requirement to simulate over extended time scales, recent studies for the S-protein are difficult to reach to µs-scale.

The computational simulations reveal more details and track more trajectories, applying in multiple fields and works (Papaleo, 2015; Ostrowska et al., 2019; Mahmood et al., 2021; Zhang and Huang, 2021). Our work uses all-atom molecular dynamics (MD) simulation and understands its characteristics at the atomic level critically. We conduct µs-scale simulations at 3°C (of a cold supply chain), 20°C (typical room temperature), 37°C (normal human body temperature), 60°C, 70°C, 80°C, and 95°C (temperatures selected to locate the critical temperature). The simulations are long enough to collect conformational samples of the S-protein for applying statistical analysis to study the effect of virus temperature stability. The analysis is based on a variety of measurements from different perspectives, including the root-mean-square deviation (RMSD), the number of hydrogen bonds (H-bonds), the solvent-accessible surface area (SASA) of the whole protein, and the mass deviation of the RBD. We also correlate the *in vitro* data of the virus life scale with the conformational change of the S-protein. Additionally, the analysis of residue based root-mean-square fluctuation (RMSF) and structural divergence are performed to isolate the temperature-sensitive residues at given temperatures.

The RBD is buried and inaccessible to receptors in a close state. The open state is necessary for binding with the receptors and to be infectious (Vankadari and Wilce, 2020). The different states of S-protein RBD, including open, close, and intermediate states, give the different potential targets for vaccination and therapeutic development (Lan et al., 2020). MD modeling has been used to investigate the binding characteristics of SARS-CoV-2; however, long-timescale MD simulations to study the temperature influence on the S-protein’s closed state conformation are uncommon. The recent work focuses on the open state of S-protein and reveals the S-protein structure details effect by 0°C–60°C in 100 ns (Khan et al., 2022). Therefore, our work with longer simulations (reach 3 μs) and a wider range of temperatures (3°C–95°C) exposes the closed state of S-protein 6VXX.PDB (Walls et al., 2020) trajectory and multi-granularity analysis, providing new insights into the RBD closed state influenced by temperature.

Our simulations of µs-long atomic resolutions have demonstrated the S-protein’s conformational changes at seven well-chosen temperatures. We discovered the existence, and the possible critical temperature, of a phase transition that rives the states of infectivity and non-infectivity. The temperatures are clustered by unsupervised learning based on an *in silico* measurements combination, and the clustering result agrees well with published *in vitro* data. Finally, the spike opening, represented by the deviation of the RBD (Zimmerman and Bowman, 2021), at different temperatures are compared. The simulations in this work are conducted on the most powerful IBM supercomputers to help achieve a fast and accurate quantitative understanding of the new virus structures to complement the sparse and inconsistent *in vitro* experiments with high-infectious virus cultivation.

## 2 Methods and materials

### 2.1 Molecular dynamics parameters

Our MD experiments are conducted on the AiMOS supercomputer, a heterogeneous system of IBM POWER9 CPUs and Nvidia V100 GPUs. The S-protein data is obtained from the protein data bank [PDB: 6VXX (Walls et al., 2020)]. CHARMM27 is employed to describe the system of the S-protein and the SPC/E water molecules. The initial structure of the S-protein 12 nm^3^ × 13 nm^3^ × 16 nm^3^ was immersed in a cubic water box of 21 nm^3^ × 21 nm^3^ × 21 nm^3^. The periodic boundary condition is applied to all three boundaries of the water box. The total number of atoms is 8,05,218, of which 45,156 (5.6%) are for the S-protein and 7,60,047 (94.4%) for water. Simulations are performed using energy minimization by gradient optimization and the canonical (NVT) ensemble. The charge neutrally is achieved by adding 15 Na^+^ to the solvent to neutralize the −15 charge in protein. During the MD simulations, S-protein coordinates are recorded every 0.1 ns and finally reach 3 μs.

### 2.2 Data collection and analysis

**FIGURE 1:**
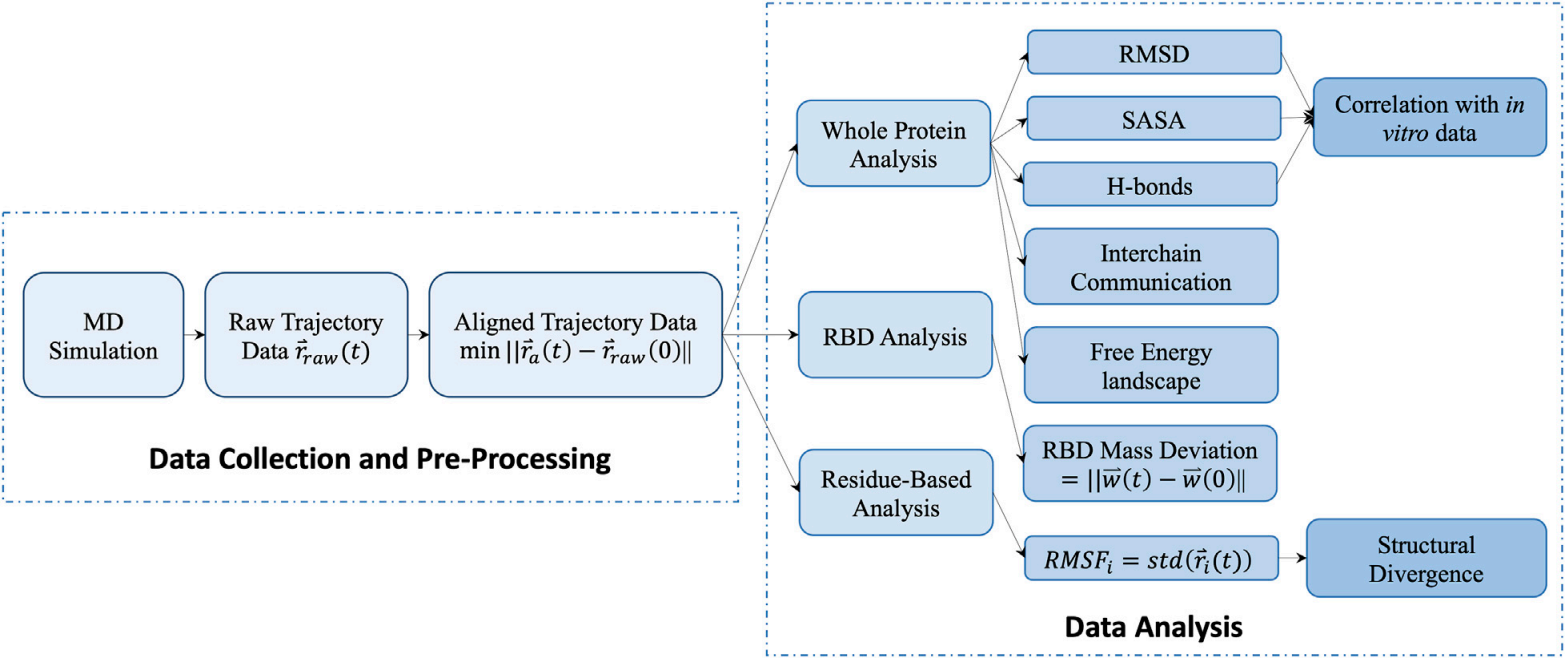
Data processing pipeline from collection to analysis.

We process the raw trajectory obtained from the simulation by aligning the protein backbone in the center of box and rotating and translating the protein backbone. The processed trajectories of atomic positions support the data analysis in three-level. 1) The protein level calculation measures the S-protein’s thermal stabilities, including the backbone RMSD, the number of mainchain-mainchain (M-M) and protein-water (P-W) H-bonds, and SASA. The unsupervised learning method K-means was performed based on those measurements to cluster different temperatures into groups of viral survival durations. The interchain communication and the free energy landscape (FEL) are also considered in the protein level calculation. 2) The domain level of the RBD mass deviation was calculated to compare the spike opening between temperatures. 3) The residue level RMSF and structural divergence were applied to measure the fluctuations of individual residues and catch the culprit residue clusters. The flowchart outlines of our data analysis are shown in Figure 1.

#### 2.2.1 Statistical analysis

To obtain a comprehensive conclusion of the temperature impact, we performed a statistical analysis based on multi-perspective measurements. The *in silico* data are used as inputs of an unsupervised learning method based on K-means to cluster different temperatures into four groups of viral survival durations, second, minute, day, and week-long survival durations accordingly. In this, the *in silico* data include the average, standard deviation, kurtosis, and skewness of RMSD, as well as the average and variation of the P-W H-bonds.

#### 2.2.2 The free energy landscape

The FEL provides insight into the S-protein conformational changes and the thermodynamics properties of the S-protein folding process. The FEL, a measurement calculated for the protein stability level, uses the deep valley to represent the lowest energy stable states and the boundaries between deep valleys to represent the intermediate conformations (Hoang et al., 2004; Khan et al., 2020; Khan et al., 2022).

The first two principal components (PC1 and PC2) are used to calculate the FEL by:

$$▵F\left(\text{PC}1,\text{ PC}2\right)=-k_{B}T\ln P\left(\text{PC}1,\text{ PC}2\right)$$

where $k_{B}$ is the Boltzmann constant, *T* denotes temperature, and P(PC1, PC2) is the normalized joint probability distribution for PC1 and PC2. The PC1 and PC2 are calculated as the projection of the last 500 ns trajectory on the eigenvectors. The eigenvectors are diagonalized and calculated from the covariance matrix, which considers the carbon alpha coordinates for S-protein.

#### 2.2.3 Conformational changes of RBD analysis

The opening is quantified in terms of how far the center of mass of an RBD (Arg319-Phe541) deviates from its position in the close (or down) state (Zimmerman and Bowman, 2021). Therefore, the RBD movements is measured by

$$D_{i}=\sqrt{{\left(x_{i}-x_{0}\right)}^{2}+{\left(y_{i}-y_{0}\right)}^{2}+{\left(z_{i}-z_{0}\right)}^{2}}$$

where $D_{i}$ is the deviation of RBD at frame i . $\left(x_{i},y_{i},z_{i}\right)$ are the coordinates of the center of mass of an RBD at frame i , and $\left(x_{0},y_{0},z_{0}\right)$ is the center of mass of an RBD at the first frame (closed state) in the simulation.

#### 2.2.4 Structural divergence analysis

The RMSF measures the time averaged root mean squared fluctuation for each residue. Structural divergence, is used to measure the residue-based structure conformational changes between two comparing temperatures T and $T_{0}$

$$SD_{i}\left(T,\;T_{0}\right)=R_{i}\left(T\right)\log\frac{R_{i}\left(T\right)}{R_{i}\left(T_{0}\right)}$$

where $R_{i}\left(T\right)$ is the RMSF at temperatures T for residue i , and $T_{0}$ is the reference structure (37°C at our cases). The logarithm measures the relative (structural) divergence of a given residue at two different conditions (temperature, in our case). Simply, if $SD_{i}\left(T,\;T_{0}\right)>0$ , the residue i deviates more in the condition T , instead of the reference structure $T_{0}$ . This measure enables us to single out residues that are responsible for the overall structural change.

## 3 Results and discussions

**FIGURE 2:**
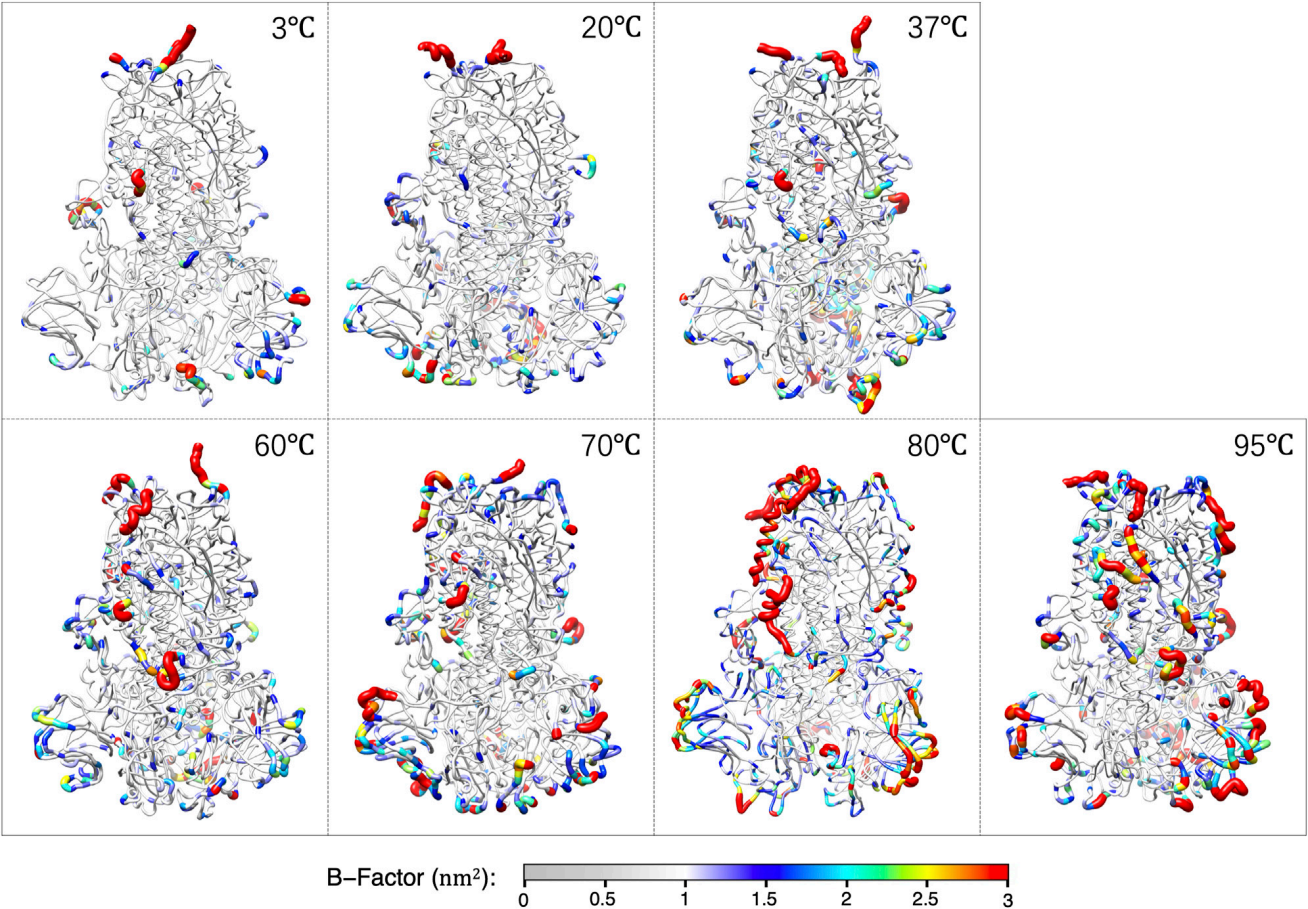
The averaged structures over the last 0.5 μs of S-protein colored by the B-factors at each temperature.

The average structures of S-protein within the last 0.5 μs for each temperature are shown in Figure 2. The color and thickness of the structure are scaled by the B-factor related to the mean square isotropic displacement of the residues. The residue with a high B-factor belongs to the flexible structure, while the low B-factor represents the well-ordered structure.

### 3.1 The protein based

#### 3.1.1 The RMSD

**FIGURE 3:**
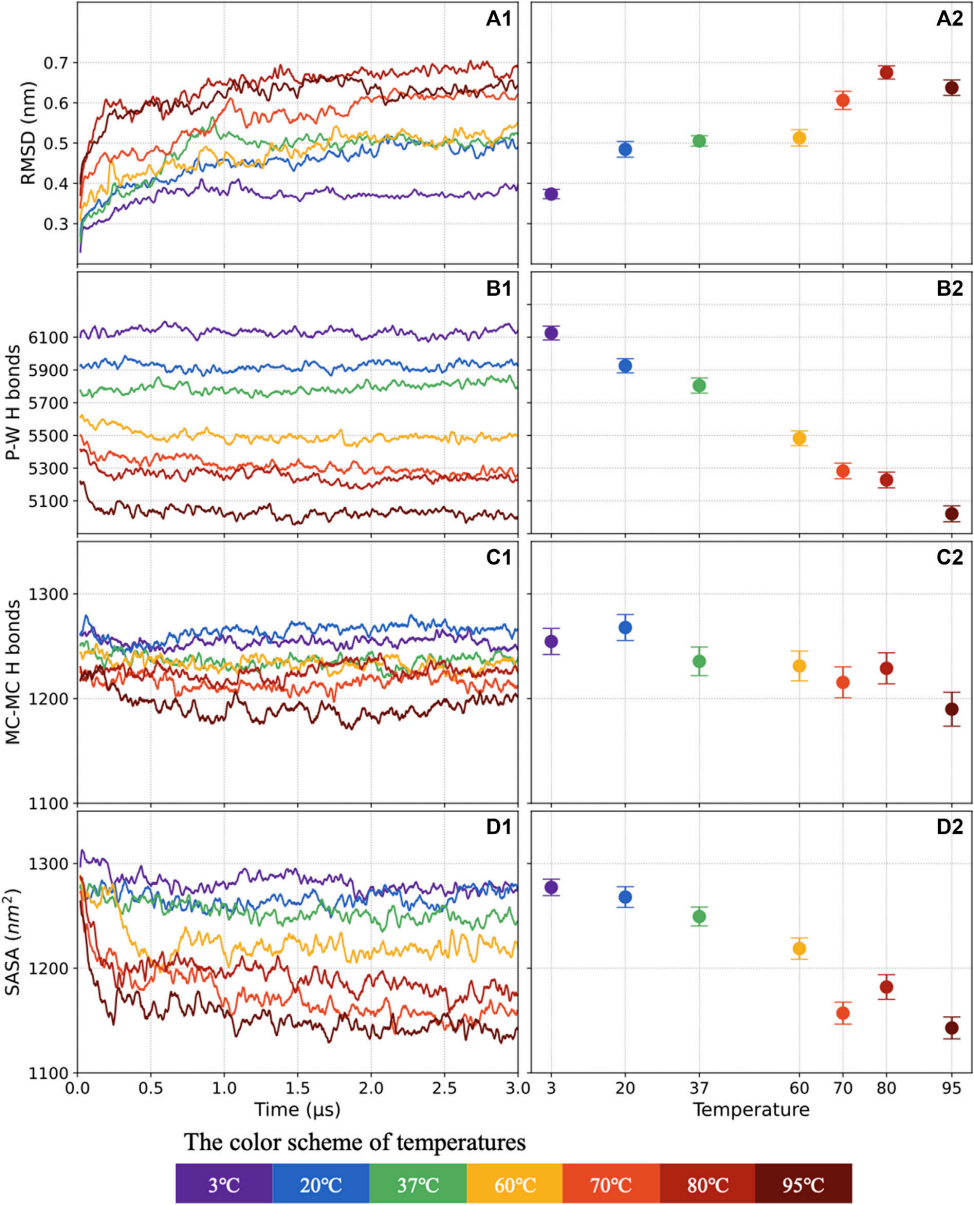
The RMSD evolving as a function of time **(A1)** and average distribution of the last 1.5 μs values **(A2)**. Similar plots show the number of P-W H-bonds **(B1,B2)**, the numbers of M-M H-bonds **(C1,C2)**, and the SASA **(D1,D2)**. This manuscript will keep using the color scheme for 3°C–95°C.

The evolution of the protein structure was monitored along the MD trajectory by calculating the average RMSD of backbone atoms taking the initial structure as the reference frame. The RMSD moving average with 0.02 μs window size is plotted against the simulated time in Figure 3A1. The mean and standard deviation of the 1.5–3 μs RMSD is shown in Figure 3A2. The S-protein shows very stably with a small RMSD (0.37 nm) at 3°C, and the RMSD gradually increases by ∼35.1% to 0.5 nm at 20°C–60°C. The S-protein becomes more unstable, with the RMSD increasing to 0.6–0.7 nm at 70°C–95°C.

The S-protein could be possible to stay long-term stable at cold temperatures with lower RMSD compared with room temperature (the RMSD at 20°C is 32.4% higher than that at 3°C). The *in silico* result is consistent with the reported case that salmon-attached coronavirus remains infectious for more than 1 week at 4°C (Dai et al., 2020). At 4°C, the virus is still stable with only ∼0.7-log reduction with infectious titers after 14 days (Chin et al., 2020). Furthermore, the S-protein becomes more sensitive to higher temperatures after 60°C. In particular, there appears a noticeable jump at RMSD from 0.53 nm (60°C) to 0.62 nm (70°C) that may serve as a signal for the presence of a critical heat denaturation around 60°C. The laboratory results (Chin et al., 2020) also showed a similar conclusion: the viral inactivation time is greatly reduced to 5 min when the incubation temperature increase to 70°C.

#### 3.1.2 The number of H-bonds

In general, the H-bond shows a high consistency with the system stability. A well-ordered protein structure tends to have more H-bond, and when the structure becomes active, the number of H-bond will decrease. The P-W and M-M H-bond counts versus simulation time and average distribution, including standards deviation, are presented in Figures 3B1–C2, respectively.

The average number of P-W H-bonds decreases quite linearly by 22.1%, from 6,126 to 5,021, as the temperature increases from 3°C to 95°C. However, the effect of increasing temperatures from 3°C to 80°C on the mainchain is insignificant. Compared with 1,254 M-M H-bonds at 3°C, only around 25 M-M H-bonds are broken, decreasing by 2%, at 80°C.

#### 3.1.3 The SASA

The SASA of S-protein explores the solvent-accessible conformational change. The SASA with 0.02 μs window size moving average are plotted in Figure 3D1, and the average distribution with standards deviation are shown in Figure 3D2. The average of SASA keeps decreasing by 10% as the temperature increases from 3°C to 95°C. The SASA shows a consistent trend with the number of P-W H-bonds, revealing that access to the solvent is a precondition to forming the H-bonds between water and protein.

#### 3.1.4 Correlating *in silico* measurements with virus’s survival times

**FIGURE 4:**
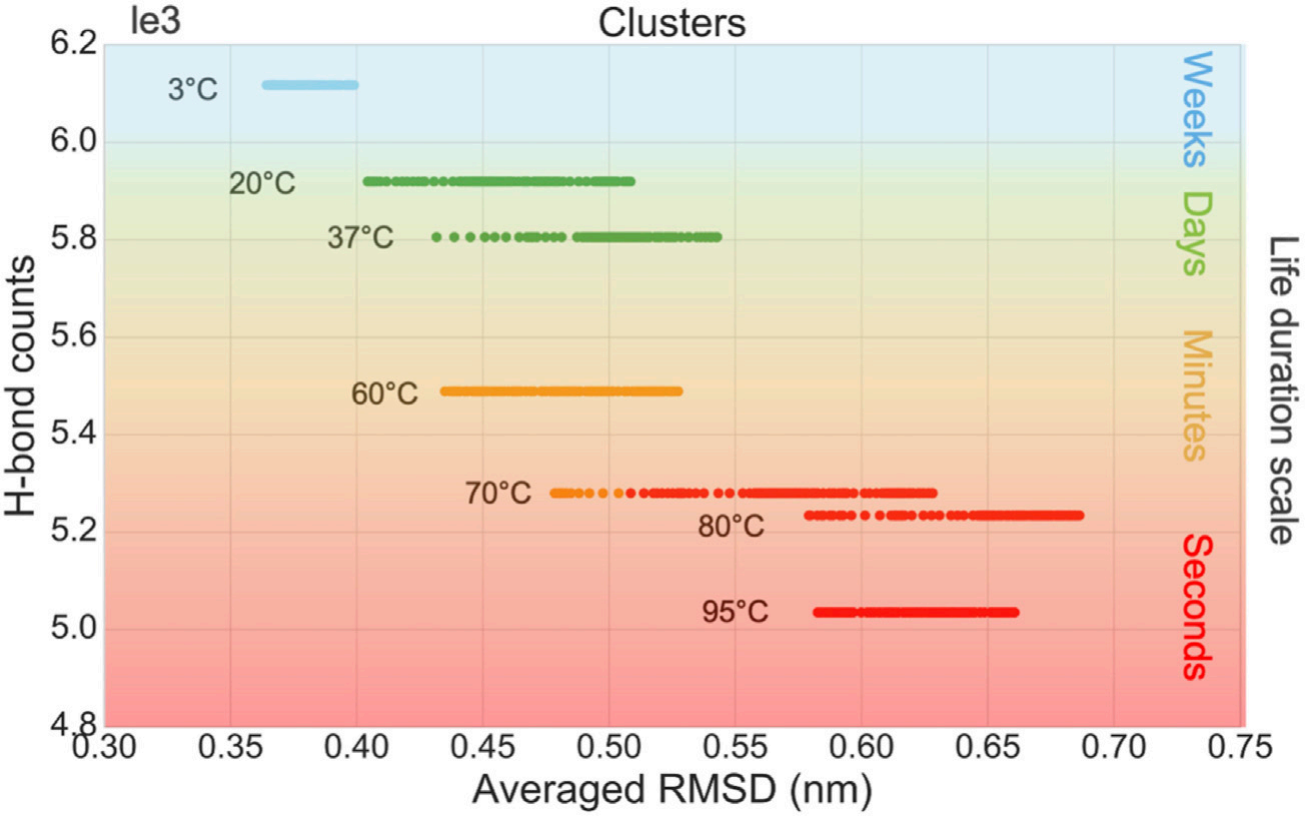
Clustering results of K-means using H-bond counts and average RMSD.

The *in silico* data are used as inputs of an unsupervised learning method to cluster different temperatures into different groups of viral survival durations. The samples are clustered by K-means, in terms of the virus life duration, based on six features, including the average, standard deviation, kurtosis, and skewness of RMSD, as well as the average and variation of the P-W H-bond counts. A moving window statistic is used with a window size of 100 ns and a stride size of 10 ns. To compare with *in vitro* data, we correlate four categories as second, minute, day, and week-long survival durations for the cluster *n* = 4 in the K-means method. The survival times of the virus at different incubation temperatures were reported by *in vitro* experiments (Chin et al., 2020). The samples are grouped into four clusters which are colored green, blue, orange, and red, shown in Figure 4 and described in Table 1.

**TABLE 1:** Clustering of survival times based on conformational information of the S-protein.

Group	Temperatures	Features descriptions
Group 1 (cold)	3°C–4°C	Blue group (Figure 4): weeks of survival time. *In vitro* data: the virus at 4°C can survive for more than 2 weeks (Chin et al., 2020).
Group 2 (normal)	20°C–37°C	Green group (Figure 4): day-scale survival time. *In vitro* data: 7 days at 20°C and 1 day at 37°C (Chin et al., 2020).
Group 3 (high)	60°C–70°C	Orange group (Figure 4): minute-scale survival time. *In vitro* data: 10 min at 56°C and around 1 min at 70°C (Chin et al., 2020).
Group 4 (very high)	Above 70°C–95°C	Red group (Figure 4): second-scale survival time. No *in vitro* data at this scale.

#### 3.1.5 The interchain communication

**FIGURE 5:**
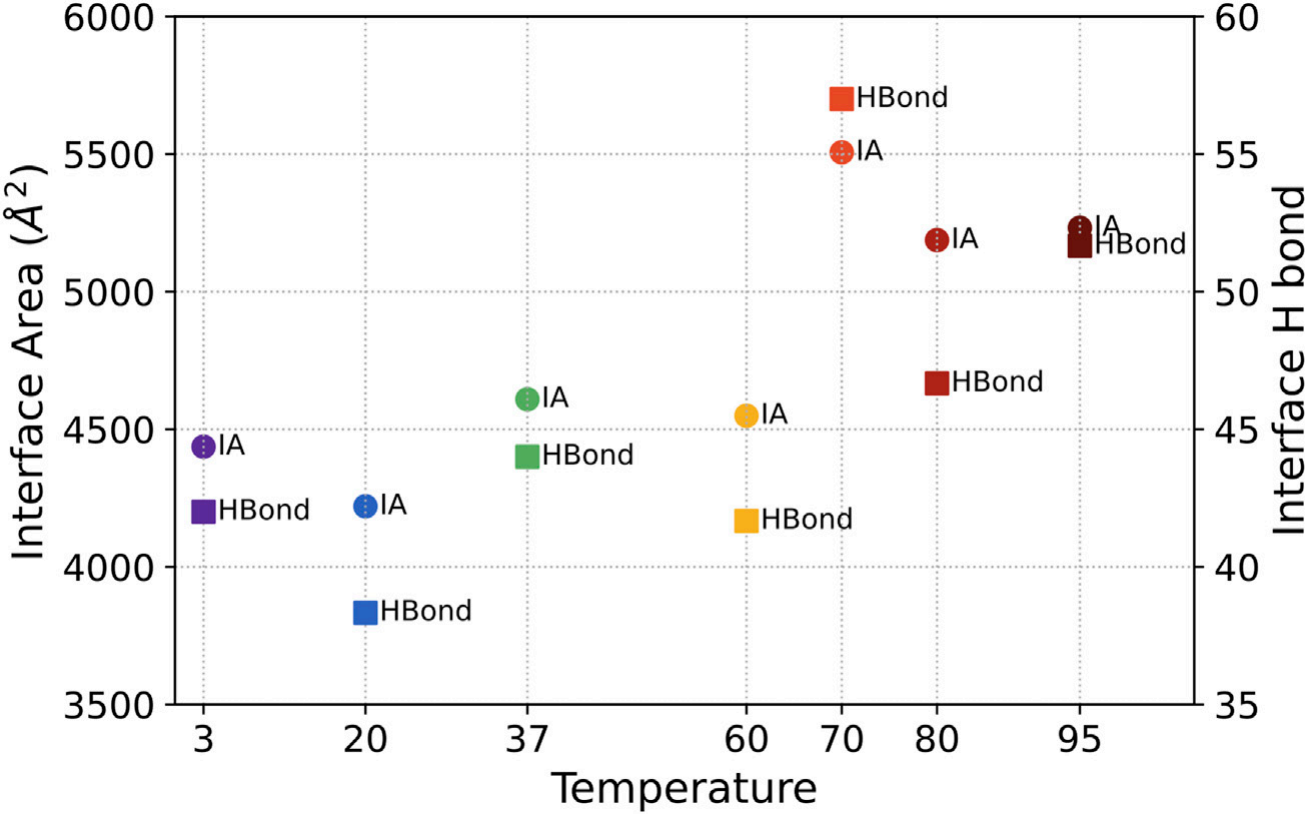
The averaged interface area (marked as IA) and the number of H-bond across the interface (marked as Hbond) between three chains for S-protein from 3°C to 95°C.

The interface area and the number of H-bond across the interface are calculated by PDBePISA (Krissinel and Henrick, 2007) and shown in Figure 5 to reveal the interaction between the three chains of S-protein. No significant change of S protein from 3°C to 60°C was detected in the interfacial area and interfacial H-bonds. The interfacial area and the interfacial H-bonds increase in the structurally unstable high temperatures from 70°C to 95°C (with higher RMSD and lower H-bonds). Therefore, instead of splitting the S protein, the interchain interactions will increase when the S protein begins to denature due to increased temperature.

#### 3.1.6 The free energy landscape

**FIGURE 6:**
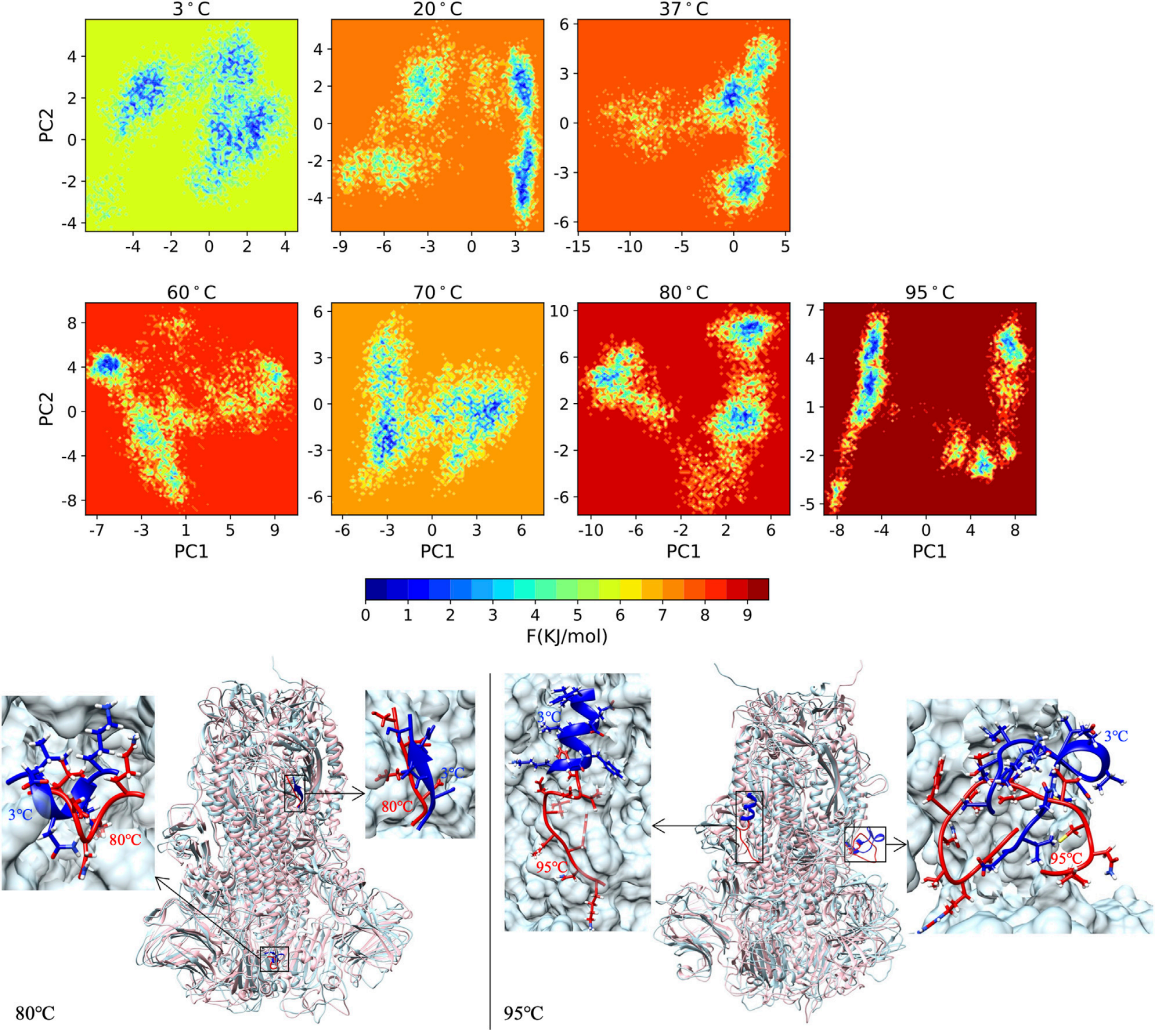
The FEL of S-protein from 3°C to 95°C with same color scale, and the protein structure for minimum energy area at 80°C (left) and 95°C (right) compared with the 3°C (blue) structure with zoomed unfolding structure.

The FEL demonstrating the energy stability of S-proteins at 3°C–95°C is shown in Figure 6. All seven temperatures are plotted with the same color scheme, and the minimum energy area is colored blue. The 3°C shows a deep valley for global energy minima while the 80°C and 95°C reveal several different energy minima states. The global blue areas describe more global stability; however, more extra blue areas indicate more transitions in the protein conformation with the thermodynamically new favorable states. We catch the protein conformation for minimum energy area at 80°C (left) and 95°C (right) compared with the 3°C (blue) structure, with zoomed unfolding structure.

The averaged potential energy, entropy, and isochoric molar heat capacity ( $C_{v}$ ) for the system are also calculated in the simulations. The details of those data are listed in Table 2. The averaged potential energy, and entropy increase with rising temperature while the $C_{v}$ decreases. The increasing entropy contributes to the S-protein thermal unfolding (Fitter, 2003; Dagan et al., 2013; Cui et al., 2014; Li et al., 2021). The $C_{v}$ decrease shows that the contribution of atomic interactions to $C_{v}$ decreases with increasing temperature, and the total kinetic energy fluctuations increase faster than that of the total potential energy (Umirzakov, 2020).

**TABLE 2:** The averaged potential energy, entropy, and isochoric heat capacity for each temperature.

Temperature (°C)	Potential energy ( $\times 10^{7}\;\text{kJ}/\text{mol}$ )	Entropy [ $\times 10^{4}\;\text{J}/\left(\text{K mol}\right)$ ]	C_v_ [ J/(K mol) ]
3	−1.25	2.51	102.02
20	−1.22	2.78	98.41
37	−1.19	3.01	97.03
60	−1.16	3.34	92.41
70	−1.14	3.39	90.09
80	−1.12	3.59	89.77
95	−1.10	3.82	86.78

### 3.2 The domain-based

#### 3.2.1 Conformational changes of RBD analysis

**FIGURE 7:**
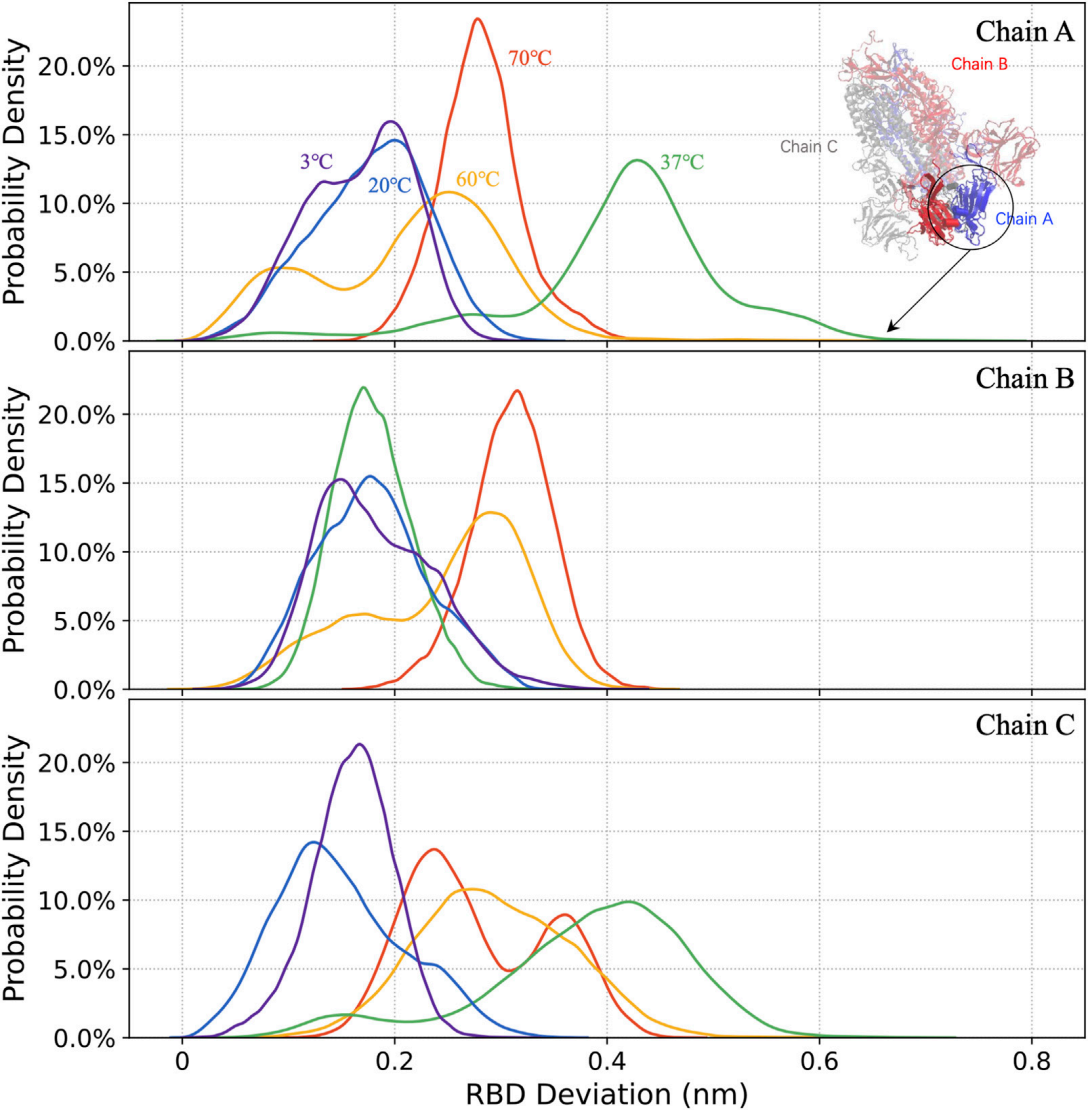
The distribution of RBD deviations in chains A, B, and C (from top to bottom) at 3°C, 20°C, 37°C, 60°C, and 70°C.

The distributions of the 1.5–3 μs RBD mass deviations for all three chains, marked by the colors of the curves as usual (Figure 3), are shown in Figure 7. The 37°C S-protein with a large RBD deviation trajectory is caught in the simulation and demonstrated in Figure 7.

The S-protein is more likely to adopt open structures at 37°C, compared to other temperatures, with the deviation of the RBD up to 0.8 nm. For 37°C, the RBD in chain A shows an active state rather than being buried by NTD, which may provide a hint for the virus infections. At 3°C, the virus that can survive for over 2 weeks (Chin et al., 2020) is less infectious. The *in silico* result: at this temperature, the small RBD deviations of 0.15 nm implied that RBD became hardly accessible to receptors for most of the time.

### 3.3 The residue-based analysis

#### 3.3.1 The RMSF

**FIGURE 8:**
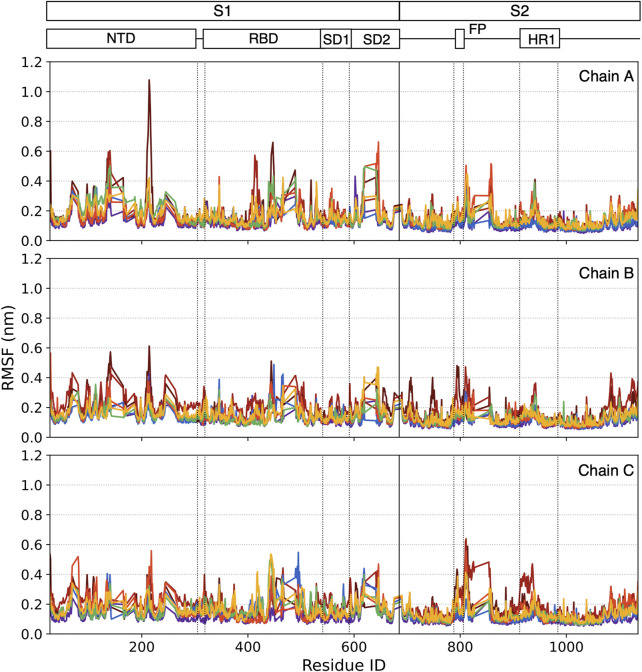
RMSF for each residue on three chains within the last 0.5 μs simulation.

RMSF is a measurement of residue fluctuation in the S-Protein. Although most residues stay in a stable state with lower RMSF, some peak residues can be easily caught. In our case, the RMSF is calculated in the last 0.5 μs time window. In Figure 8, each point represented the RMSF of a specific residue ordered by its residue ID and colored by the color scale mentioned in Figure 3.

In the lower temperature group 3°C–20°C, no significant residue has RMSF larger than 0.5 nm. In the higher temperature group (37°C and 60°C), some individual residue RMSF reaches 0.5 nm. For 70°C, more residues have higher RMSF, especially the N643-R646 cluster in Chain A, have RMSF larger than 0.5 nm. More residue clusters are active in the very high-temperature range.

At 80°C, some residue clusters’ average RMSFs are higher than 0.5 nm, including C136-L141, G413-K417 in Chain A, and C662-I664 in Chain C. At 95°C, the RMSFs of I210-Q218 and K444-N448 in Chain A, F140-G142, and R214-D215 in Chain B, as well as A27-Y28 and S810-K814 in Chain C are larger than 0.5 nm.

#### 3.3.2 The structural divergence

**FIGURE 9:**
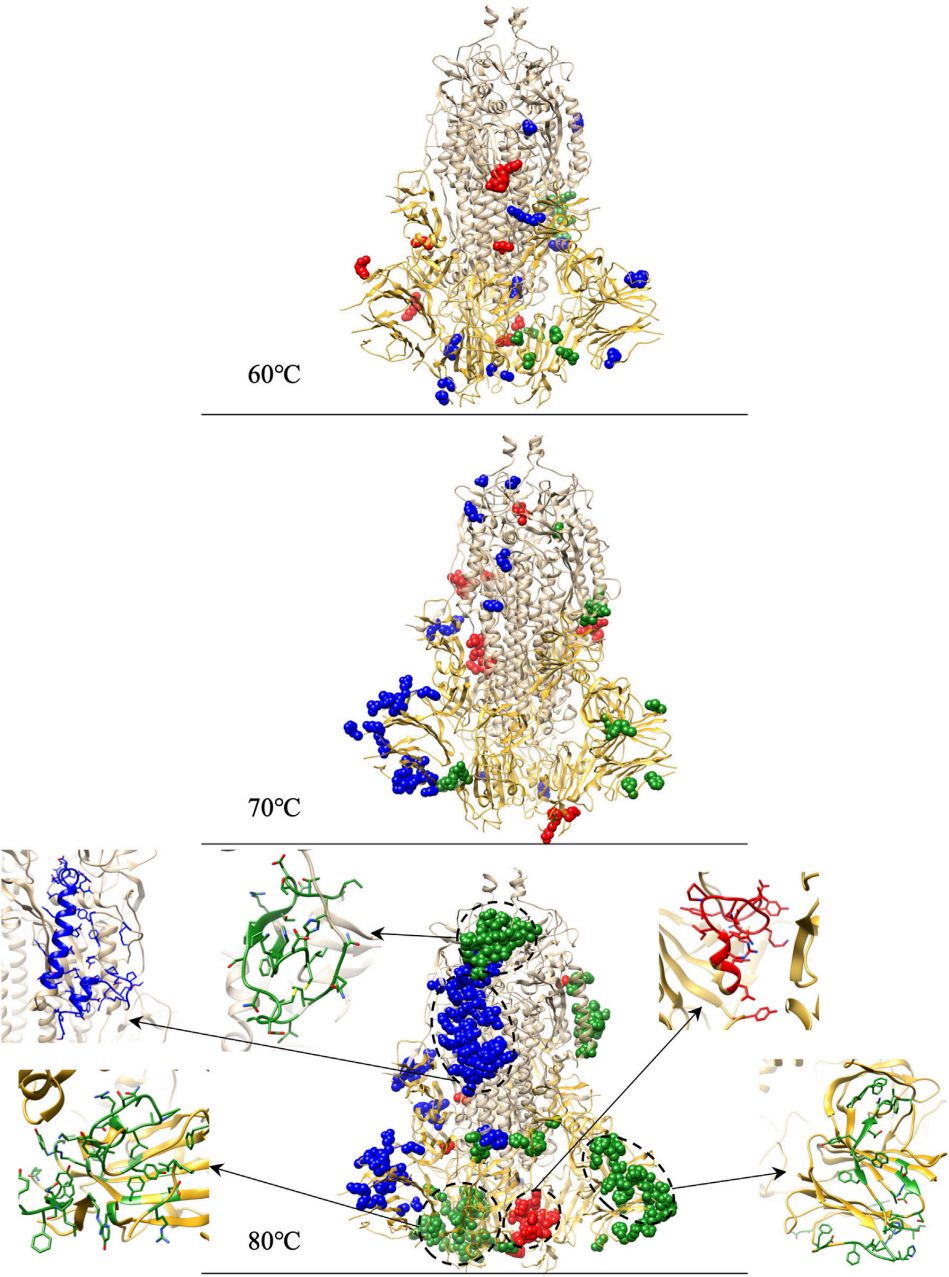
Identifications of residue outliers causing protein structure divergences and the residue outlier clusters zoomed structure for 80°C.

We calculate the structural divergence in the interesting temperature zone of 60°C–80°C. To spot the outliers of residues that cause significant structure divergence, we set $SD_{i}\left(T,\;T_{0}\right)=0.07$ as the threshold with which we found the outliers for 60°C–80°C listed in Table 3. The specific positions of the residue distribution in the protein, with zoomed outliers’ clusters for 80°C, are shown in Figure 9. The dark yellow represents the S1 domain, and the light yellow represents the S2 domain. The cluster structure marked with balls are residue outliers, and its red, green, and blue colors represent chain A, B, and C, respectively.

**TABLE 3:** Structural divergence outliers list.

Temperature	Chain	Residue ID
60	A	R214 F318 R408 T415 K529 S530 P812-R815 L858
	B	Y369 T415 I418 F464 R466 N616 E619 V620 N641 V642 Q644-R646
	C	F133 T208-I210 A372 S375 K417-A419 Y449 Y451 G504 T645 R646 V705 K795 F855 R995
70	A	Y451 F497 Q498 F643-A647 S810-K811 S813-K814 F817 F855-L861 T912 N914
	B	A27 Y28 K41 F135 F140 L212 R357 R466 I468 Y489 R815 F823 K825 V826 Y904
	C	H69 D80 N81 K97 T108 L110-Ser112 T114 Q115 F133-F135 C166 I210-V213 D215-F220 S371 Y495 F643 T645 R646 K795 K811 Y1110 D1118 N1135
80	A	C136-N137 D405 E406 R408-Y423 Y505 S704 T747 G858
	B	A27-N30 F32 T33 F58-S60 W64 F65 A67 H69 T95 E96 S98 I100 N122-T124 F135 D138 F140-Val143 N165 Y170 R190 F220 L244 H245 A263 T315 N317-R319 Y351 S371 A372 D398 F400 R403 R408-I410 A411-A419 Y421 Y423 F429 A435 R454 K462 R466 Y489- F497 N501 V503-Y505 Y508 V512 D568-D571 F592 T791-P793 P807- F817 D820 N824 K825 I1081- F1089 R1091 I1114-I1130 I1132-N1135
	C	A27 T29 L84 P85 N87 K97 S98 I100 G107 T108 I210 N211 D215- F220 N234 T240 Y269 F318 R319 F374 I468 G545 F592-G594 V642-R646 V705 K795 G799 F800 F802 Q804 P807-K811 K814-T827 K854-N856 T912-L938 Q965

At 60°C (11, 13, and 18 outliers in chain A, B, and C, respectively) and 70°C (22, 15, and 34 outliers in chain A, B, and C, respectively), outliers are evenly distributed in the entire S-protein. At 80°C, among the 131 outliers in chain B, 45 are concentrated in RBD, 40 out of 45 forming a cluster zoomed in Figure 9. Moreover, 34 out of the 131 outliers in chain B are concentrated in NTD, and 33 out of 34 constitute a cluster shown in Figure 9. 51 out of 91 outliers form a cluster in chain C and are concentrated on the carbon chain of S2. We capture a large number of outliers in 80°C forming clusters considered as the culprit residues that caused 80°C RMSD higher than other temperatures.

## 4 Conclusion

Our simulations of µs-long atomic resolutions demonstrate the S-protein’s conformational changes affected by temperature varying and enable us to infer the existence and the possible critical temperature for the S-protein. In the whole protein-based, domain-based, and residue-based analysis, we could get: 1) The S-protein of SARS-CoV-2 is structurally stable at 3°C with the lowest RMSD, the highest number of H-bonds, and the stable RBD. Even in the residue-based analysis, no residue can be caught for large RMSF. 2) All the measurements point out a consistent signal that the heat effect difference between 60°C and 70°C is significant, especially in the RMSD gap and outlier residue clusters catch in the structure divergence analysis. 3) In extremely high temperatures (80°C and 95°C), the S-protein is most active with the highest RMSD and lowest number of H-bonds; moreover, the RMSF provides evidence for the culprit residue that leads S-protein to denature in the short-term. More detailly, in µs-scale, 80°C are broken first in the RBD, NTD, and carbon chain of the S2 domain in the structure divergence analysis.

Our *in silico* results could be correlated with the published *in vitro* results to correlate ambient temperature with the life duration and infectivity of the virus. 4) The temperatures from 3°C to 95°C are clustered into four groups by an unsupervised learning algorithm, and the clustering result agrees with *in vitro* viral survival time scale from weeks to seconds (Chin et al., 2020) (Figure 4). 5) The RBD mass deviation is large at 37°C while it is low at cold temperature (3°C), room temperature (20°C), and high temperatures (higher than 60°C). This indicates the S-protein is more likely to adopt an open structure at 37°C, although the life duration of the virus is shorter than it at 3°C.

For the first time, we present the µs-scale MD studies of the temperature-varying conformation of a life-threatening S-protein. Our simulations on the most powerful IBM supercomputers achieve a fast and accurate quantitative understanding of the new virus structures and corroborate well with the published *in vitro* experiments. Moreover, our simulations complement the effect of a wide range of temperature on closed state S-protein.

One bottleneck of the current all-atom simulation is the computing power, including computing resources and algorithms. To improve the simulation speed, a multiscale model concurrently considering components at their own characteristic scales (Zhu et al., 2021) and an intelligent time-stepping algorithm (Han et al., 2021) can effectively relieve the computing load and shorten the simulation time. Advances on machine learning based techniques also have been made towards intelligent image processing for simulation parameter determination (Zhang et al., 2021b; Sheriff et al., 2021) and dynamics prediction (Zhang et al., 2021a), which enables long-term study with affordable efforts.

## Data Availability

The original contributions presented in the study are included in the article/Supplementary Material, further inquiries can be directed to the corresponding author.
